# Lysine acetylation of DosR regulates the hypoxia response of *Mycobacterium tuberculosis*

**DOI:** 10.1038/s41426-018-0032-2

**Published:** 2018-03-21

**Authors:** Hua Yang, Wei Sha, Zhonghua Liu, Tianqi Tang, Haipeng Liu, Lianhua Qin, Zhenling Cui, Jianxia Chen, Feng Liu, Ruijuan Zheng, Xiaochen Huang, Jie Wang, Yonghong Feng, Baoxue Ge

**Affiliations:** 1 Shanghai Key Laboratory of Tuberculosis, Clinic and Research Center of Tuberculosis, Shanghai Pulmonary Hospital, Tongji University School of Medicine, Shanghai, 200433 PR China; 2 Department of Microbiology and Immunology, Tongji University School of Medicine, Shanghai, 200049 PR China

## Abstract

Tuberculosis caused by *Mycobacterium tuberculosis* (*Mtb*) infection remains a large global public health problem. One striking characteristic of *Mtb* is its ability to adapt to hypoxia and trigger the ensuing transition to a dormant state for persistent infection, but how the hypoxia response of *Mtb* is regulated remains largely unknown. Here we performed a quantitative acetylome analysis to compare the acetylation profile of *Mtb* under aeration and hypoxia, and showed that 377 acetylation sites in 269 *Mtb* proteins were significantly changed under hypoxia. In particular, deacetylation of dormancy survival regulator (DosR) at K182 promoted the hypoxia response in *Mtb* and enhanced the transcription of DosR-targeted genes. Mechanistically, recombinant DosR^K182R^ protein demonstrated enhanced DNA-binding activity in comparison with DosR^K182Q^ protein. Moreover, Rv0998 was identified as an acetyltransferase that mediates the acetylation of DosR at K182. Deletion of Rv0998 also promoted the adaptation of *Mtb* to hypoxia and the transcription of DosR-targeted genes. Mice infected with an *Mtb* strain containing acetylation-defective DosR^K182R^ had much lower bacterial counts and less severe histopathological impairments compared with those infected with the wild-type strain. Our findings suggest that hypoxia induces the deacetylation of DosR, which in turn increases its DNA-binding ability to promote the transcription of target genes, allowing *Mtb* to shift to dormancy under hypoxia.

## Introduction

In 2015, ~10.4 million new infections and 1.8 million deaths globally were caused by *Mycobacterium tuberculosis* (*Mtb*)^1^. The remarkable success of *Mtb* as a pathogen is due to its marked phenotypic drug resistance and its ability to evade the host immune system. *Mtb* can also enter into a nonreplicating state and persist in humans for extended periods without causing disease in what is known as a latent tuberculosis (TB) infection (LTBI)^2^. Clarification of the mechanisms underlying the LTBI state may lead to the development of useful pharmaceuticals to prevent *Mtb* infection or even cure individuals harboring *Mtb* in the dormant stage^3^. Adaptations to hypoxia have been implicated to play a prominent role in the dormancy of *Mtb*^4^. Accumulating evidence has demonstrated that oxygen tension correlates tightly with *Mtb* growth, the formation of hard, fibrous, hypoxic granulomas, and, eventually, the development of infection^5^. Wayne et al.^6, 7^ have pioneered the development of culture conditions that gradually deprive bacteria of oxygen to generate nonreplicating persistent bacilli in vitro as a model for latency. With this, Schubert et al.^8^ profiled the proteome of dormant *Mtb* and observed that the stress-induced dormancy survival regulator (DosR) regulon consisted of ~20% of the cellular protein content during dormancy. However, whether posttranslational modifications (PTMs) are involved in this process remains elusive.

N^ε^-lysine acetylation is an abundant and evolutionarily conserved PTM found in prokaryotes and eukaryotes^9^. As a dynamic and reversible process, lysine acetylation affects protein conformation and/or charge, thus altering DNA-binding affinity, enzymatic activity, protein–protein interactions, and protein or mRNA stability^10, 11^. Increased global analyses of lysine acetylation in bacteria have been reported, including in *Escherichia coli*^12, 13^, *Erwinia amylovora*^14^, *Bacillus subtilis*^15^, *Salmonella enteric*^16^, and *Mtb*^17, 18^, as well as in others^19^. Protein acetylation is implicated in nearly all cellular processes, such as central metabolism, protein translation, and pathogen virulence^20, 21^. Increasing evidence has shown that protein acetylation plays an important regulatory role in mycobacteria^22, 23^. The universal stress protein (USP) was the first-characterized acetylated protein in mycobacterium^24^. Recently, Bi et al.^25^ identified the modulation of central carbon metabolism by the acetylation of isocitrate lyase in *Mtb*. Given the widespread regulatory role of lysine acetylation, it is tempting to speculate that lysine acetylation is involved in the hypoxia adaption of *Mtb*. Hence, we examined alterations in the protein acetylation profile of *Mtb* in response to hypoxia by quantitative proteomics and subsequently identified 377 acetylation sites in 269 proteins that were significantly changed. Furthermore, we confirmed the lysine acetylation of DosR and its role in the response of *Mtb* to hypoxia.

## Results

### Quantitative proteomic profiling of lysine acetylation in *Mtb* H37Rv under aeration and hypoxia

**Fig. 1:**
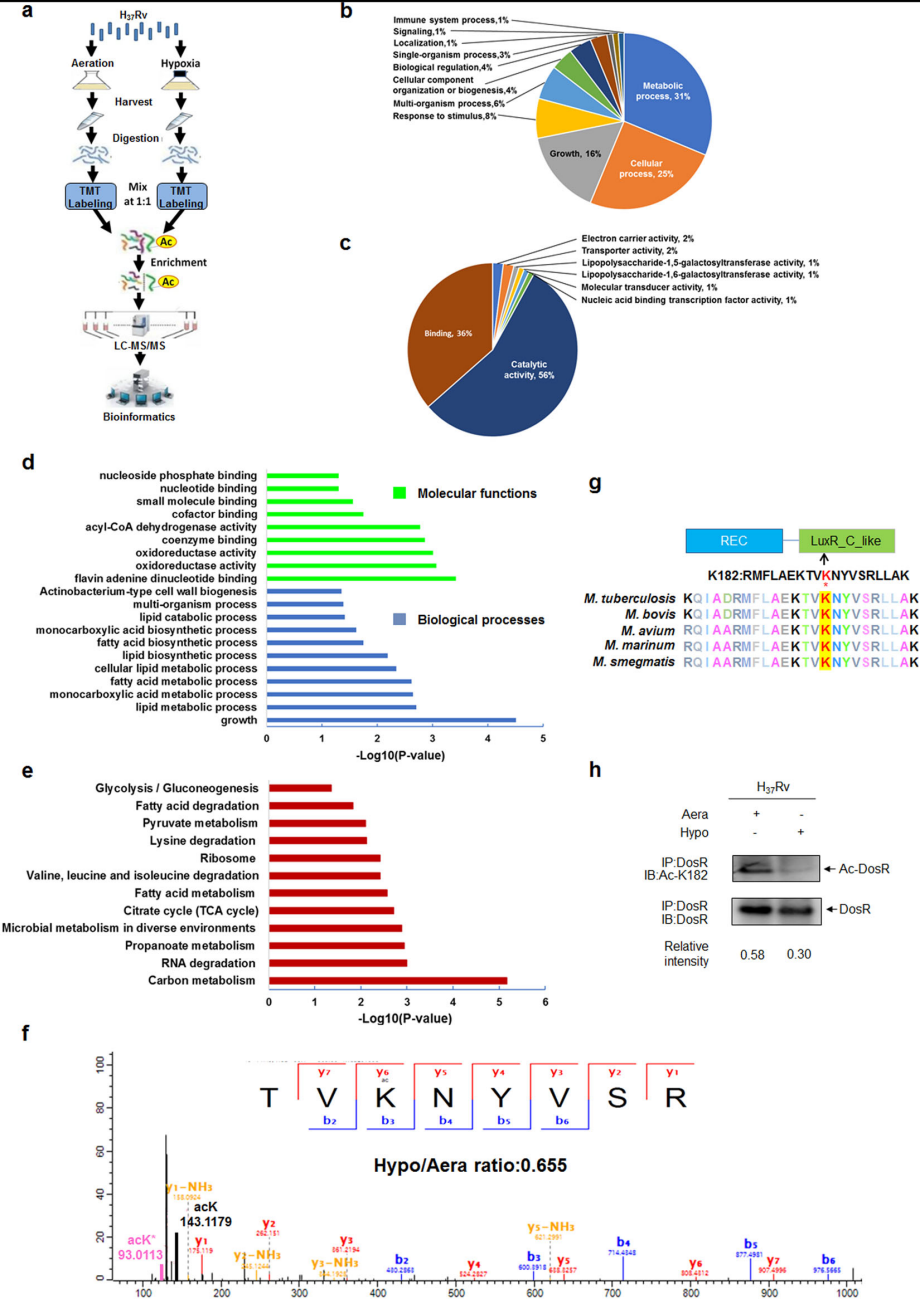
Hypoxia induces deacetylation of DosR. **a** Workflow of quantitative proteomics analysis of lysine acetylation in H_37_Rv under hypoxia. *Mtb* H_37_Rv was cultured under aeration or hypoxia in conical screw-capped Nephelo flasks with 20 mm side arms according to the Wayne model. Samples for aeration (Aera) and hypoxia (Hypo) cultures were harvested after 12 days. Proteins were extracted and digested by trypsin. After separate labeling with a tandem mass tag (TMT) 6-plex kit, peptides from Aera or Hypo were mixed 1:1, enriched with anti-acetyl lysine antibody beads and loaded for LC–MS/MS analysis. The resulting MS/MS data were processed for bioinformatics analysis. **b**, **c** Gene ontology functional classification of biological processes (**b**) and molecular functions (**c**) of 215 upregulated acetylated proteins under hypoxia. **d** Gene ontology enrichment analysis of biological processes and molecular functions of 215 upregulated acetylated proteins under hypoxia. The differently colored bars indicate the corrected *p* values for the enrichment of the annotations. **e** KEGG pathway enrichment analysis of 215 upregulated acetylated proteins under hypoxia. **f** Mass spectroscopy (MS) analysis identified K182 acetylation of DosR. **g** K182 in DosR is conserved among various mycobacteria species. The asterisk denotes the conserved lysine residue, and this result was analyzed by DNASTAR software. **h** Acetylation of DosR^K182^ in H_37_Rv WCL under hypoxia was downregulated. H_37_Rv were grown under aeration or hypoxia in 7H9 broth supplemented with 10% ADC for 3 days. DosR proteins were immunoprecipitated (IP) by the anti-DosR antibody. The acetylation level of DosR was determined by immunoblotting (IB) using an anti-DosR Ac-K182-specific polyclonal antibody. Western blots are representative of at least three independent experiments

To profile the lysine acetylation of *Mtb* during exponential growth and hypoxia-induced dormancy, we cultured *Mtb* H_37_Rv under normal aerobic conditions and gradually decreasing oxygen levels using the Wayne model (Supplementary Figure S1)^26^. We harvested cultures at day 12 during exponential growth and hypoxia. Acetylated peptides were enriched using a specific acetyl lysine antibody and analyzed by liquid chromatography–tandem mass spectrometry (LC–MS/MS) (Fig. 1a). We identified 1215 acetylation sites in 679 proteins, of which 852 acetylation sites in 497 proteins were quantified (Supplementary Table S1). Relative quantitation was divided into two categories: under hypoxia, 321 acetylation sites in 215 proteins were upregulated; and 56 acetylation sites in 54 proteins were downregulated (Supplementary Table S2). Classification results for biological processes and molecular functions both showed that the largest group of upregulated acetylated proteins under hypoxia consisted of enzymatic proteins associated with metabolism, which accounted for 31% and 56% of the upregulated acetylated proteins, respectively (Fig. 1b, c). These findings are consistent with the previous observation that most lysine-acetylated proteins are involved in metabolism.

Furthermore, we performed Gene Ontology (GO) enrichment analysis on the acetylation data. As revealed previously, hypoxia induces comprehensive changes in the bacterium, with *Mtb* shifting to exploit lipids as the primary nutrient source^27^. Consistently, our data demonstrated that upregulated acetylated proteins under hypoxia were enriched for biological processes related to lipid metabolism (Fig. 1d). From the GO enrichment analysis, we also found that upregulated acetylated proteins under hypoxia were significantly enriched for some specific molecular functions, such as flavin adenine dinucleotide binding, oxidoreductase activity, and coenzyme binding (Fig. 1d). For the 54 acetylated proteins downregulated under hypoxia, biological processes associated with the growth of a symbiont involved in its interaction with a host were found to be enriched (data not shown). Additionally, proteins with ligase activity that form carbon–nitrogen bonds also had a higher tendency to be acetylated under normal aerobic conditions. For the Kyoto Encyclopedia of Genes and Genomes (KEGG) metabolic pathways analysis, upregulated acetylated proteins under hypoxia were highly enriched for carbon metabolism, RNA degradation, and propanoate metabolism, among others (Fig. 1e), while acetylated proteins involved in the ribosome were highly enriched under aerobic conditions. Overall, these data indicate that lysine acetylation might play a comprehensive regulatory role in the adaption of *Mtb* to hypoxia.

### Hypoxia induces deacetylation of DosR

Numerous studies have demonstrated the important role of DosR during the physiological adaptation of *Mtb* to hypoxia^28, 29^. Three amino acids in DosR, namely, Lys179, Lys182, and Asn183, directly bind to nucleotide bases in the DNA motif and induce the expression of target genes^30^. Mutation of Lys182 directly reduces the DNA-binding affinity of DosR and abrogates the induction of its regulon genes, thus highlighting the important role of K182 in DosR^31;^ however, the relevant mechanism is still unclear. From our quantitative acetylome profiling data, DosR was shown to be acetylated at K182 (Fig. 1f). Considering the location and positive charge of this lysine residue, we hypothesized that acetylation of K182 may be involved in regulating DosR activity, specifically the DNA-binding ability of DosR. Sequence alignment analysis demonstrated that K182, the acetylated lysine residue, is evolutionarily conserved among different mycobacteria species (Fig. 1g). Interestingly, quantitative proteomics analysis revealed that the acetylation of DosR K182 was reduced under hypoxia. To further confirm whether the acetylation levels of DosR K182 respond to hypoxic stress, we produced anti-DosR- and anti-DosR Ac-K182-specific polyclonal antibodies (characterized as shown in Supplementary Figure S2) and applied these to examine the acetylation levels of DosR K182 in *Mtb* H_37_Rv cultured under normal aerobic or hypoxic conditions. Consistently, a significant reduction of acetylation at K182 of DosR under hypoxia was observed (Fig. 1h), indicating that DosR may undergo deacetylation at K182 in response to hypoxia.

### Deacetylation of DosR promotes the adaption of *Mtb* to hypoxia

**Fig. 2:**
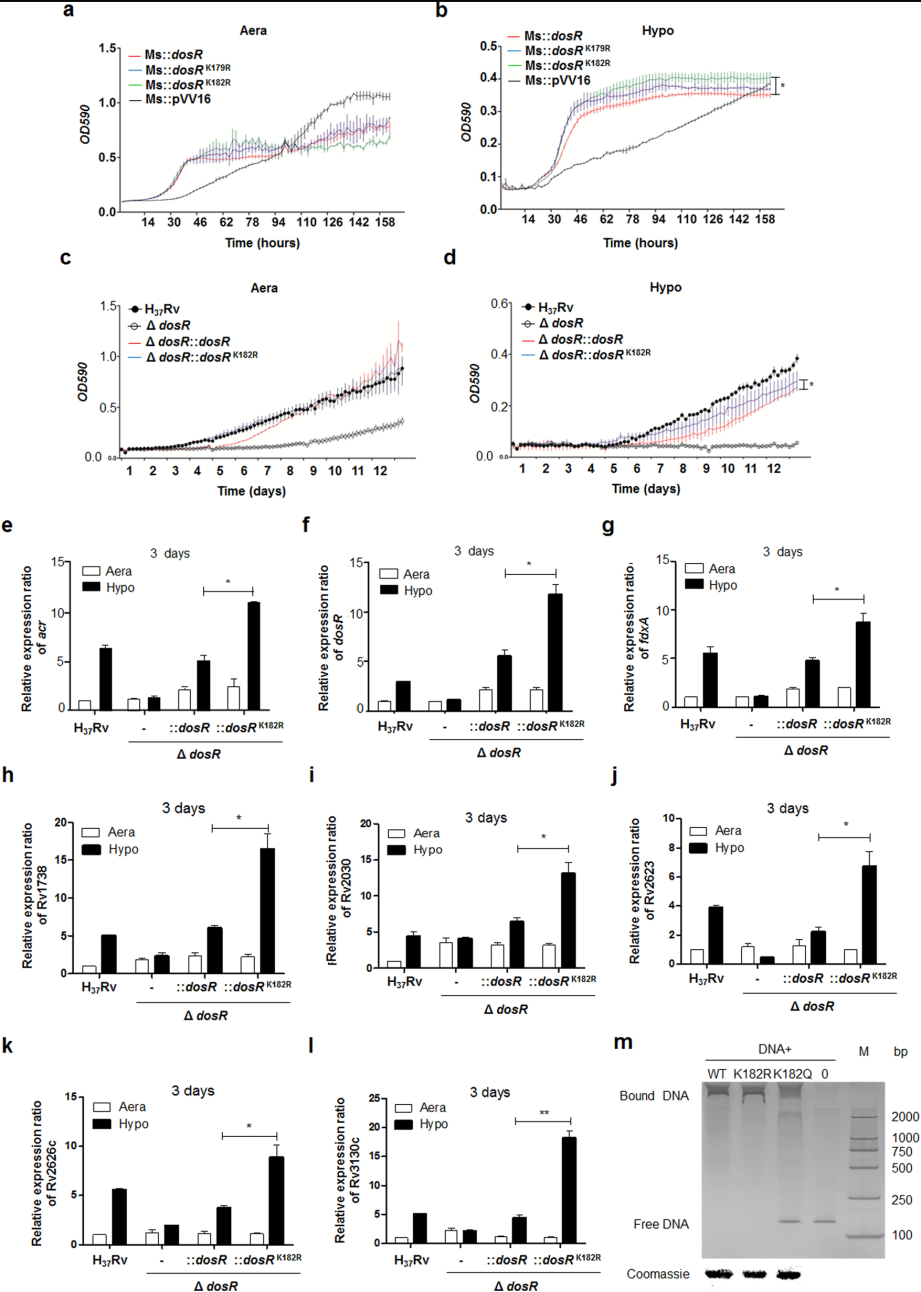
Deacetylation of DosR promotes the hypoxia adaption of *Mtb*. **a**, **b** Growth in aeration (**a**) or hypoxia (**b**) of *M. smegmatis* overexpressing DosR or a K-R mutant. Recombinant *M. smegmatis* overexpressing DosR or the K-R mutant were grown to mid-log phase; the growth curve was measured using a Bioscreen Growth Curve Instrument. *M. smegmatis* harboring pVV16 was used as a control. Hypoxic conditions were established by covering bacterial suspensions with paraffin oil. The optical density was measured at an absorbance of 590 nm every 2 h. Cultures were grown at 37 °C for 7 days. The results were combined (mean ± standard deviation (SD)) from two independent experiments, with each experiment performed in triplicate. A statistically significant difference was found between Ms::*dosR* and Ms::*dosR*^K182R^ under hypoxic conditions, as analyzed by Student’s *t*-test (*P < 0.05). **c**, **d** Growth under aeration (**c**) or hypoxia (**d**) of H_37_Rv, the deletion mutant of *dosR*, and WT or K182R complement mutants. Growth curves were measured as described above, and cultures were grown at 37 °C for 14 days. A significant difference was also found between the WT complement strain and K182R complement mutants under hypoxia. *P < 0.05. Student’s *t*-test. **e**–**l** The K182R mutant promoted the transcription of the target genes *acr* (**e**), *dosR* (**f**), *fdxA* (**g**), Rv1738 (**h**), Rv2030 (**i**), Rv2623 (**j**), Rv2626c (**k**), and Rv3130c (**l**). Bacteria were cultured and harvested at day 3 to isolate total RNA. Relative transcriptional levels were determined using the 2^−ΔΔCt^ method. The reference gene used was 16S rRNA. Values represent the mean ± SD from three independent experiments. *P < 0.05. ** *P*<0.01. Student’s *t*-test. **m** Deacetylation of K182 strengthened the DNA-binding activity of DosR. An electrophoretic mobility shift assay (EMSA) was used to test the binding of DosR and the derivative mutant proteins K182R and K182Q to the promoter region of *acr*. The EMSA result is representative of at least three independent experiments

Based on the quantitative acetylome data generated in this study, six of the key regulated proteins from the hypoxia regulatory network^27^ were acetylated at various levels in response to hypoxic stress, namely, DosR, PhoP, SigA, SigB, SigF, and Lsr2 (Table 1). To examine whether acetylation affects the bacterium’s adaption to hypoxia, we generated recombinant *Mycobacterium smegmatis* expressing corresponding wild-type (WT) genes, as well as counterparts with a lysine-to-arginine substitution of the identified acetylated lysine to mimic the non-acetylated form. The effect of deacetylation on the growth of 13 recombinant *M. smegmatis* strains under hypoxia was then analyzed. We found that DosR K182R and SigA K334R mutants have significant effects on growth under hypoxia in *M. smegmatis* (Fig. 2a, b and Supplementary Figure S3), which suggests that the adaption to hypoxia by mycobacteria may be regulated by the lysine acetylation of DosR or SigA. Furthermore, we constructed *Mtb* H_37_Rv mutants deficient in DosR (H_37_Rv:Δ*dosR*) (characterized as shown in Supplementary Figure S4A–S4C), complemented with WT DosR (H_37_Rv:Δ*dosR*::*dosR*) and deacetylation mutants of K182 (mimicked by an arginine substitution, K182R; H_37_Rv:Δ*dosR*::*dosR*^*K182R*^). In line with the observations of recombinant *M. smegmatis*, the deacetylation mutant did not have a significant effect on normal aerobic growth but significantly promoted the response to hypoxia by *Mtb* (Fig. 2c, d), suggesting that acetylation of DosR K182 directly regulates the adaption of *Mtb* to hypoxia.

**Table 1:** Acetylation of six regulated proteins from *Mtb* hypoxia regulatory network

Protein	Protein description	Position	Hypo-vs-Aera
P71814	OmpR family two-component system response regulator	197	3.47
P9WGI3	RNA polymerase sigma factor SigF	121	2.45
P9WGI5	RNA polymerase sigma factor SigB	308	2.21
P9WGI1	RNA polymerase sigma factor SigA	334	1.57
P9WMF9	Transcriptional regulatory protein DevR (DosR)	179	1.53
		182	0.65
P9WIP7	Nucleoid-associated protein Lsr2	43	0.53

### Deacetylation of K182 regulates target gene transcription and the DNA-binding activity of DosR

DosR controls the dormancy survival regulon that consists of ~50 genes^32^. Since deacetylation of DosR K182 directly promotes *Mtb* adaption to hypoxia, we hypothesized that acetylation of DosR K182 regulated the transcription of dormancy survival-related genes. Several known DosR-regulated genes, including *acr*, *dosR*, *fdxA*, Rv1738, Rv2030, Rv2623, Rv2626c, and Rv3130c, were selected to evaluate whether K182 acetylation affects the activity of DosR as a transcription factor. A DosR deletion mutant of *Mtb*, WT, and a deacetylation complementation mutant were cultured under hypoxic conditions as described previously. As expected, the transcription levels of eight candidate genes were all significantly upregulated for the K182R mutant under hypoxia compared with normal aeration conditions and the WT strain (Fig. 2e–l). Furthermore, we wanted to determine whether acetylation of K182 directly hindered the DNA-binding activity of DosR. We constructed expression plasmids of WT DosR and its derivatives, K182R and K182Q (substituted with glutamine to mimic the constitutively acetylated form), using a 6× His tag sequence at their C terminals. All proteins were overexpressed, purified, and adjusted to a similar concentration. electrophoretic mobility shift assay (EMSA) was performed by incubating the above purified DosR derivatives with the *acr* promoter DNA region, followed by non-denaturing polyacrylamide gel electrophoresis and silver staining analysis. EMSA showed that K182Q had almost no DNA-binding affinity compared to WT DosR, while K182R had a similar DNA-binding ability to WT DosR (Fig. 2m). Therefore, acetylation of the K182 residue is critical for the DNA-binding ability of DosR, the transcription of DosR downstream target genes, and the growth of *Mtb* under hypoxic conditions.

### Rv0998 acetylates DosR and negatively regulates the adaption of *Mtb* to hypoxia

**Fig. 3:**
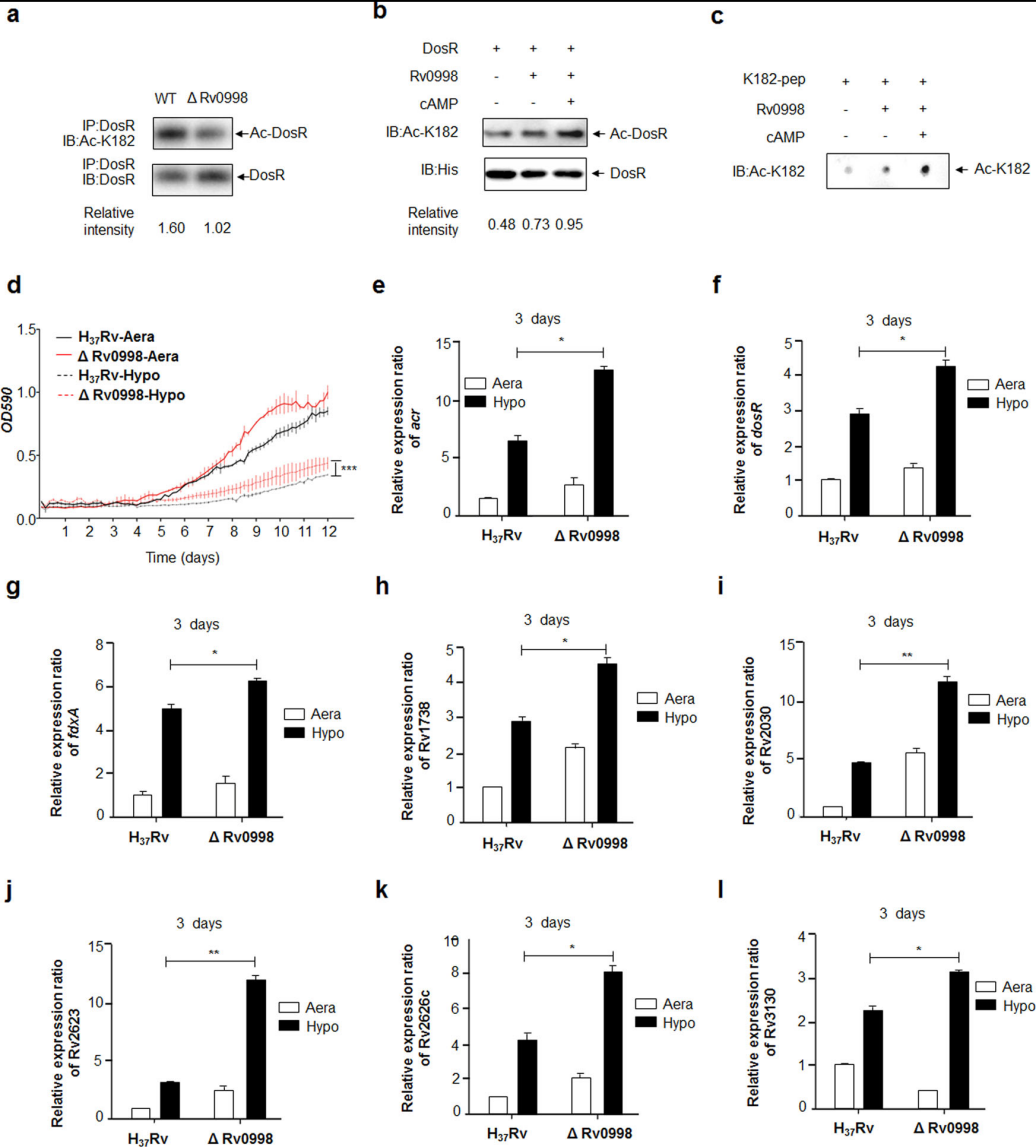
Rv0998 acetylates DosR and negatively regulates the adaption of *Mtb* to hypoxia. **a** Acetylation of DosR^K182^ in the Rv0998 deletion mutant of *Mtb* H_37_Rv was downregulated. DosR from the Rv0998 deletion mutant or the wild-type *Mtb* H_37_Rv was immunoprecipitated (IP) by an anti-DosR antibody. Acetylation levels were detected by immunoblotting (IB) with an anti-DosR Ac-K182-specific polyclonal antibody. Western blots were repeated at least three times—a representative blot is shown. **b** Rv0998 acetylates DosR K182 in vitro. DosR (2 μg) was incubated with or without Rv0998 (2 μg) or cAMP (200 μM). Acetylation levels were determined by immunoblotting. The western blot shown is representative of at least three independent experiments. **c** Rv0998 acetylates K182 peptides of DosR in vitro. K182 peptide (2 μg) was incubated, with or without Rv0998 (2 μg) or cAMP (200 μM). Acetylation levels were determined by dot blot. The dot blot shown is representative of at least three independent experiments. **d** Deletion of Rv0998 promoted the hypoxia response of *Mtb*. A growth curve was measured for the Rv0998 deletion mutant and wild-type *Mtb* H_37_Rv under aeration and hypoxia. **e**–**l** Deletion of Rv0998 promoted the transcription of *acr* (**e**), *dosR* (**f**), *fdxA* (**g**), Rv1738 (**h**), Rv2030 (**i**), Rv2623 (**j**), Rv2626c (**k**), and Rv3130c (**l**). Bacteria were cultured and harvested on day 3 to isolate total RNA. Relative transcriptional levels were determined using the 2^−ΔΔCt^ method. The expression of tested genes was normalized to that of 16S rRNA. Values represent the mean ± SD from three independent experiments. *P < 0.05.** *P*<0.01. Student’s *t*-test

Lysine acetylation is reversible and is performed in bacteria by members of the GCN5-related *N*-acetyltransferase (GNAT) superfamily, as well as by NAD^+^-dependent deacetylases^33^. In *Mtb*, 20 GNATs have been predicted. The cAMP-dependent protein lysine acetyltransferase Rv0998 was recently shown to catalyze the acetylation of many important proteins^34^. To examine whether Rv0998 catalyzes the acetylation of DosR K182, we constructed an Rv0998 deletion mutant of *Mtb* H_37_Rv (characterized as shown in Supplementary Figures S4D and S4E). Western blotting revealed that the acetylation level of DosR at K182 in the Rv0998 deletion mutant was significantly reduced (Fig. 3a). To further confirm whether DosR is a substrate of Rv0998, we overexpressed and purified 6× His-tagged DosR proteins from *E. coli*, as well as Rv0998. DosR was incubated with Rv0998 in the presence of AcCoA and/or cAMP. Western blotting revealed that DosR K182 could be acetylated by Rv0998 in vitro without cAMP, although cAMP further enhanced the function of Rv0998 (Fig. 3b). We also synthesized a short peptide of DosR, from 175 to 188 amino acids in length, termed K182-pep. In vitro acetylation reactions further confirmed that Rv0998 could acetylate DosR K182-pep (Fig. 3c). Next, we used the Rv0998 deletion mutant to confirm the effect of K182 acetylation on growth under hypoxia and on target gene transcription. The Rv0998 deletion mutant was found to significantly promote the *Mtb* response to hypoxia (Fig. 3d) and to dramatically activate the transcription of eight DosR target genes (Fig. 3e–l).

### Protein acetylation contributes to the pathogenesis of *Mtb*

**Fig. 4:**
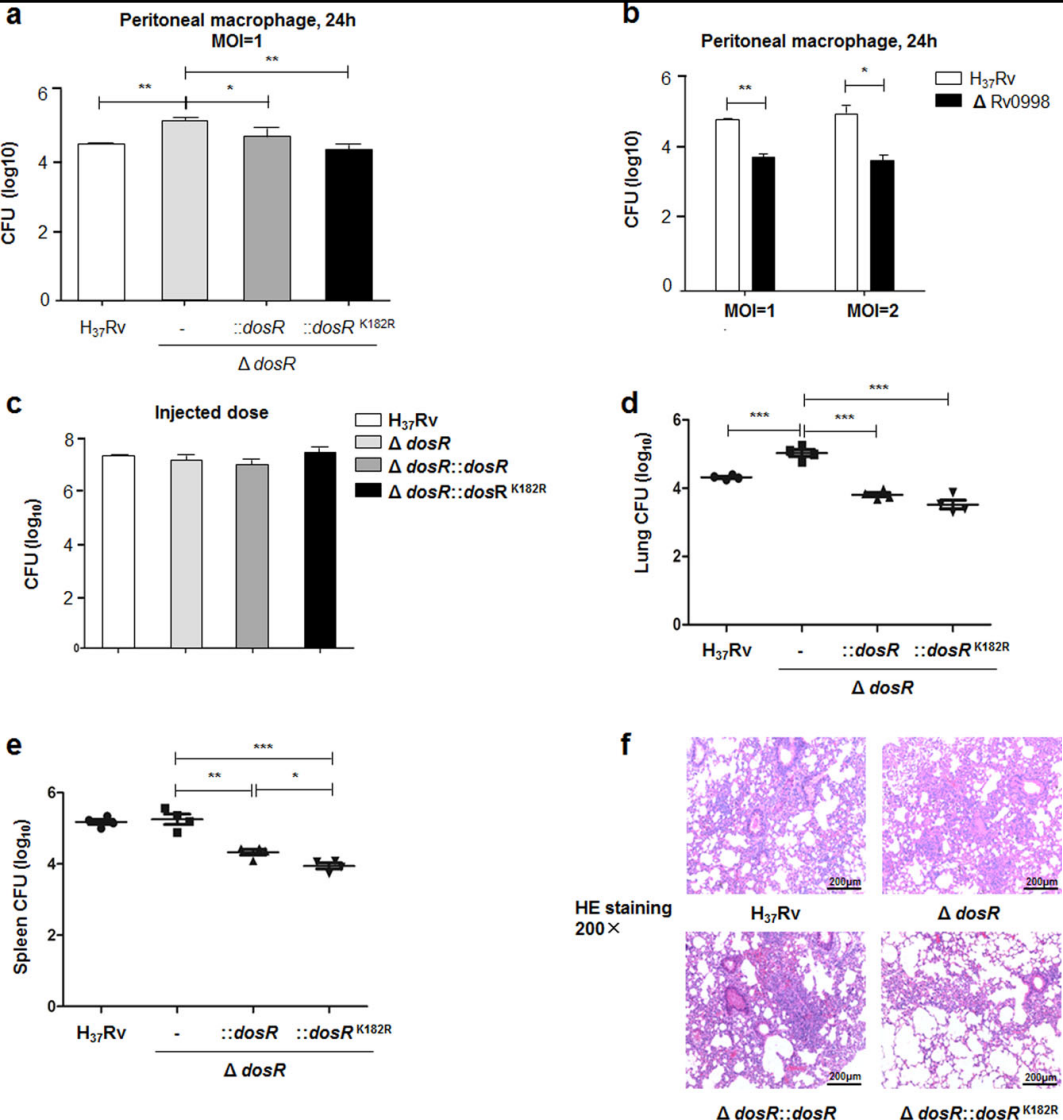
Deacetylation of DosR contributes to the intracellular survival and pathogenesis of *Mtb*. **a** Deacetylation of K182 inhibited the intracellular survival of *Mtb*. Primary peritoneal macrophages were harvested as described in Materials and Methods and infected at a multiplicity of infection (MOI) of 1 for 24 h. Infected cells were lysed, and the number of viable intracellular bacteria was determined by serial dilutions and plating on 7H10 agar plates. Values represent the mean ± SD of three independent experiments. **b** The deletion of Rv0998 inhibited the intracellular survival of *Mtb*. Peritoneal macrophages were infected at an MOI of 1 or 2 for 24 h. Bacterial colonies were counted to calculate intracellular survival efficiency. Values represent the mean ± SD of three independent experiments. **c** Doses of *Mtb* H_37_Rv, the deletion mutant of DosR, and WT or K182R complement mutants were injected into mice. The number of bacteria in the injected doses for the four bacterial groups was determined by serial dilutions and plating on 7H10 agar plates and was found to be similar for all groups. **d**, **e** Lung (**d**) and spleen (**e**) colony-forming units (CFUs) of C57Bl/6 mice infected for 28 days with *Mtb* H_37_Rv, the deletion mutant of *dosR*, and WT or K182R complement mutants. Female C57Bl/6 mice (6–8 weeks old) were challenged by intraperitoneal (i.p.) injection with 5 × 10^6^ CFU in 100 μL PBS. The left side of the lung tissues from infected mice and the whole spleen were homogenized, diluted, and plated on 7H10 agar plates. Colonies were counted after 4 weeks. **f** Lung histopathology of C57Bl/6 mice infected with *Mtb* H_37_Rv, the deletion mutant of *dosR*, and WT or K182R complement mutants for 28 days. Lung halves of mice infected for 28 days were fixed and embedded in paraffin, and then 5 μm-thick sections were stained with hematoxylin and eosin (HE) by standard methods. The pathology was evaluated by pathologists in a blinded manner. Images are pseudocolored representations at ×200 magnification. Values represent the mean ± SD from two independent experiments. *P < 0.05, **P < 0.01, ***P < 0.001. Student’s *t*-test

Earlier studies revealed that DosR plays a critical regulatory role in the adaptation and survival of *Mtb* within cells and tissues^35, 36^. We hypothesized that DosR K182 acetylation was critical for bacterial survival and proliferation in macrophages. To answer this question, primary peritoneal macrophages were infected with *Mtb* H_37_Rv, the deletion mutant of DosR, and WT or K182R complement mutants with a multiplicity of infection (MOI) of 1. After 24 h, cells were lysed, and the number of intracellular bacteria was calculated by colony-forming unit (CFU) counting. We found that the deletion mutant multiplied more efficiently than *Mtb* H_37_Rv, WT, or K182R complement mutants, in agreement with the studies of Parish^35^. The K182R mutant exhibited lower bacterial proliferation than the WT complement mutants (Fig. 4a). We also used the deletion mutant of Rv0998 to detect the intracellular survival of *Mtb* in peritoneal macrophages. As expected, we observed significantly retarded growth of the Rv0998 deletion mutant, with multiplicities of infection of 1 or 2 at 24 h (Fig. 4b).

To further understand the functional role of DosR K182 acetylation in the pathogenesis of TB, we performed intraperitoneal (i.p.) infections with *Mtb* in mice. We found that even though the average injected dose for the four groups was similar (Fig. 4c), the numbers of bacteria in the lungs and spleen were both elevated in mice infected with the DosR deletion mutant for 28 days (Fig. 4d, e). The number of DosR complement mutant bacilli in the lungs and spleen was significantly decreased compared with the deletion mutant, while that of the K182R complement mutant was even lower (Fig. 4d, e). From lung histopathological analysis, larger lesions and the pathological involvement of a greater proportion of the lung were observed for mice infected with the DosR deletion mutant relative to mice infected with DosR and K182R complement mutant bacilli, although damage by the K182R mutant was milder (Fig. 4f). In conclusion, the deacetylation of K182 caused significantly lower *Mtb* bacterial loads as well as attenuated pulmonary inflammation in a mouse model.

## Discussion

As is well known, hypoxia stress limits the in vivo growth of *Mtb*. However, *Mtb* can adapt to limited oxygen by entering into a metabolically altered state while awaiting the opportunity to reactivate. Defining the strategies employed by *Mtb* to shape its adaption to hypoxia is of intense interest. Many “omics” analyses have been used to comprehensively evaluate the response of *Mtb* to hypoxia^8, 27, 37^. The utilization of specific antibodies to acetylated peptides in immunoprecipitation has significantly improved the enrichment and identification of acetylated lysine residues with proteomic profiling^38^. Although an analysis of the acetylome of *Mtb* has recently been reported^17, 18, 25^, the impact of lysine acetylation on the adaption of *Mtb* to hypoxia has not yet been addressed. Understanding how *Mtb* modulates lysine acetylation in response to oxygen limitation may lead to new strategies for its control.

We implemented a universally applicable, tandem mass tag (TMT)-labeled approach to estimate variations in lysine acetylation in *Mtb* during exponential growth and hypoxia-induced dormancy^39^. We identified 1215 acetylation sites on 679 proteins, many more than found by Bi et al.^25^. The 852 acetylation sites on 497 proteins were identified under both aerated and hypoxic conditions and were quantified. In addition, 363 residual unquantifiable acetylation sites may have also been derived from dramatic variations in this modification under different growth conditions, or they could have been due to the poor replicability of the high-throughput analyses^40^. Among the quantified sites, we found 377 acetylation sites on 269 proteins that were significantly changed. These findings strongly indicate that protein acetylation is important for the regulation of protein function and the subsequent adaption to hypoxia. Unfortunately, the quantification experiment was performed only once (two samples and one biological replicate for each experimental condition), so there was neither a description of reproducibility nor any statistical significance. Although we lacked biological replicates, we produced many functional experiments to verify the reliability of the proteomics data. After screening by proteomics data, we selected DosR as our research object. Taking into account the reliability of the data, we produced an anti-DosR Ac-K182-specific polyclonal antibody to determine whether the acetylation of the target protein was consistent with the proteomics data. On the other hand, we produced a qualitative experiment of acetylation in the same strain of H_37_Rv. Compared with the previous study, there are many proteins with the same acetylation site in the quantitative experiment (data not shown), which also includes the K182 site of DosR. This reflects the reliability of our quantitative results.

DosR is a key transcription factor that facilitates *Mtb* adaptation to hypoxia, and K182 plays an important role in the DNA-binding function of DosR^41^. Based on the altered K182 acetylation under hypoxia as detected by quantitative proteomics, we further detected this modification using a specific polyclonal antibody to lysine acetylation. This showed that oxygen limitation stress led to the deacetylation of DosR at K182. Because of the important role of DosR in the response of *Mtb* to hypoxia, we focused on the functional and regulatory mechanism of K182 acetylation in DosR. We found that K182 deacetylation directly regulated *Mtb* growth under hypoxia with knockout and complement strains of *Mtb* H_37_Rv (Fig. 2d), in accordance with the results from recombinant *M. smegmatis*.

The acetylation of transcription factors has recently been demonstrated to be a conserved regulatory means to modulate gene expression in both bacteria and eukaryotes^42, 43^. Similarly, our results suggest that deacetylation of K182 is critical for the DNA-binding activity of DosR (Fig. 2m), which promoted the transcription of DosR downstream target genes (Fig. 2e–l), leading to the rapid response of *Mtb* to hypoxia. We subsequently attempted to identify the key acetyltransferases that regulated the reversible acetylation of DosR K182. We measured the K182 acetylation level in recombinant *M. smegmatis* overexpressing five acetyltransferases from *Mtb* and found that one lysine acetyltransferase, Rv0998, significantly promoted the acetylation of DosR (data not shown). From experiments using the deletion mutant and an in vitro acetylation assay (Fig. 3a–c), we postulated that DosR was a substrate of Rv0998. The residual acetylation of DosR in the Rv0998 deletion mutant indicated that another as yet to be identified acetyltransferase(s) is also involved in the regulation of the acetylation of DosR. The phenotype obtained from the Rv0998 deletion mutant further supported the notion that deacetylation of DosR promoted target gene transcription and had a profound effect on the growth of *Mtb* under hypoxia (Fig. 3d–l).

Of note, earlier studies revealed a contradictory role for DosR in *Mtb* virulence. Parish et al.^35^ reported that the DosR mutant of *Mtb* is hypervirulent in both SCID and C57Bl/6 mice, while studies in guinea pigs, rabbits, and even macaques revealed that mutants of DosR exhibited reduced infectivity^36, 44, 45^. Here we showed that the deletion mutant of DosR promoted the intracellular survival of *Mtb* in macrophages, while a K182R mutant exhibited lower proliferation than WT complement mutants (Fig. 4a) as well as an Rv0998 deletion mutant (Fig. 4b). In infected mice, deacetylation of K182 also caused significantly lower bacterial loads as well as attenuated lung damage (Fig. 4c–f). Although isolated macrophages and a C57Bl/6 mouse infection model may not represent the real hypoxic situation in vivo, these infection results suggest that deacetylation of K182 can promote the regulatory effect of DosR on *Mtb* virulence under different conditions. Studies in more effective infection models should be performed to further confirm our findings.

In conclusion, we propose that hypoxia may induce the deacetylation of DosR, which in turn increases its DNA-binding ability to promote the transcription of DosR downstream target genes, allowing *Mtb* to rapidly adapt to hypoxia (Supplementary Figure S5).

## Materials and methods

### Bacterial strains, plasmids, primers, and media

All strains, plasmids, and primers used in this study have been described in Supplementary Table S3 and S4. Middlebrook 7H9 broth (Difco/Becton Dickson, Franklin Lakes, NJ) supplemented with 10% ADC (5% bovine serum albumin (BSA), 2% dextrose, and 5% catalase) was used as an enriched medium. Middlebrook 7H10 agar (Difco) plates contained 10% ADC, 1.5% (w/v) agar, and antibiotic supplements as required. The antibiotics used were 100 μg/mL ampicillin, 50 μg/mL or 75 μg/mL hygromycin B, and 50 μg/mL kanamycin.

### Bacterial cultures

Experiments performed by Wayne^26^ in *Mtb* H_37_Rv have been described previously. In brief, conical screw-capped Nephelo flasks with 20 mm side arms and flat bases (Wheaton Scientific Products, Millville, NJ) were used to culture bacteria. *Mtb* H_37_Rv was grown in 7H9 broth supplemented with 10% ADC and 0.05% Tween 80 at 37 °C with shaking to mid-log phase (OD_590_ ≈ 0.4, ~2.5 × 10^8^ CFU/mL). For aerobic, fully replicating control cultures, 200 mL of medium was inoculated with 2 mL of the culture (OD_590_ of 0.4) in the flask and incubated at 37 °C on a magnetic stirrer set to rotate at 180 r.p.m. Simultaneously, 400 mL of medium was inoculated with 4 mL of identical culture in a tightly capped flask, which left 200 mL of air space, corresponding to a 0.5 head space ratio. The tightly capped flask culture was then placed on a tissue culture magnetic stirrer set at 70 r.p.m. and incubated at 37 °C to avoid perturbation of the surface. Cultures of the two flasks were gently tipped into the side arm to read OD_590_ daily. Samples for the quantitative proteomics assay were taken after 12 days of vigorous aerated culture (Aera), which then entered the platform stage. Similarly, after 12 days of hypoxic culture (Hypo), samples entered anaerobic nonreplicating persistent stage 2. Using 20 mm screw-capped culture tubes, small volumes of cultures under aeration or hypoxia were collected as samples for western blot or real-time (RT)–polymerase chain reaction (PCR) detection.

### Protein extraction, trypsin digestion, TMT labeling, and enrichment of acetylated lysine peptides

Cultured cells were harvested and washed twice with cold phosphate-buffered saline (PBS); lysed in 8 M urea supplemented with 1 mM dithiothreitol (DTT), 2 mM ethylenediaminetetraacetic acid (EDTA), 1% Protease Inhibitor Cocktail Set III (Calbiochem, San Diego, CA), and 30 mM nicotinamide; and then disrupted by sonication. Soluble proteins were collected after centrifugation at 20 000 × *g* for 10 min at 4 °C. Protein concentrations were determined with a BCA protein assay kit (Thermo Fisher Scientific, Waltham, MA) according to the manufacturer’s instructions. For trypsin digestion, the protein sample was diluted by adding 100 mM triethylammonium bicarbonate buffer (TEAB; Sigma-Aldrich, St. Louis, MI) to a urea concentration <2 M; trypsin was then added at a 1:50 trypsin-to-protein mass ratio for the first digestion overnight and at a 1:100 trypsin-to-protein mass ratio for a second 4 h digestion. After trypsin digestion, peptides were desalted using a Strata X C18 SPE column (Phenomenex, Torrance, CA) and vacuum-dried. Peptides were reconstituted in 0.5 M TEAB and labeled according to the manufacturer’s protocol for a TMT 6-plex kit (Thermo Fisher Scientific). To enrich Kac peptides, tryptic peptides dissolved in NETN buffer (100 mM NaCl, 1 mM EDTA, 50 mM Tris-HCl, and 0.5% NP-40, pH 8.0) were incubated with pre-washed antibody beads (PTM Biolabs, Hangzhou, China) at 4 °C overnight with gentle shaking. The beads were washed four times with NETN buffer and twice with ddH_2_O. Bound peptides were eluted from beads with 0.1% trifluoroacetic acid. The eluted fractions were combined and vacuum-dried. The resulting peptides were cleaned with C18 ZipTips (Millipore, Billerica, MA) according to the manufacturer’s instructions.

### LC–MS/MS analysis, database search, and bioinformatics analysis

The tryptic peptides were dissolved in 0.1% formic acid (solvent A), directly loaded onto a homemade reversed-phase analytical column (15 cm length and 75 μm inner diameter). The gradient consisted of an increase from 6 to 23% solvent B (0.1% formic acid in 98% acetonitrile) over 26 min, 23 to 35% over 8 min, an increase to 80% over 3 min, and then holding at 80% for the last 3 min, all at a constant flow rate of 400 nL/min on an EASY-nLC 1000 UPLC system. The peptides were subjected to an NSI source followed by MS/MS in Q ExactiveTM Plus (Thermo Fisher Scientific) coupled online to the UPLC. The electrospray voltage applied was 2.0 kV. The *m*/*z* scan range was 350–1800 for a full scan, and intact peptides were detected in the Orbitrap at a resolution of 70 000. Peptides were then selected for MS/MS using an NCE setting of 28, and the fragments were detected in the Orbitrap at a resolution of 17 500. A data-dependent procedure alternated between one MS scan followed by 20 MS/MS scans with a 15.0 s dynamic exclusion. Automatic gain control was set at 5E4. The fixed first mass was set as 100 *m*/*z*.

The resulting MS/MS data were processed using the Maxquant search engine (v.1.5.2.8). Tandem mass spectra were searched against an *Mtb* database concatenated with a reverse decoy database. Trypsin/P was specified as the cleavage enzyme, allowing up to two missing cleavages. The first search range was set to 5 p.p.m. for precursor ions; the main search range was set to 5 p.p.m. and 0.02 Da for fragment ions. Carbamidomethyl on Cys was specified as a fixed modification, and oxidation on Met was specified as a variable modification. False discovery rate was adjusted to <1%, and the minimum score for peptides was set to >40. For the quantification method, TMT 6-plex was selected. After checking the mass error and length of all identified peptides that fit both requirements, the relative quantitation was divided into two categories: a quantitative ratio of more than 1.5 was considered to be upregulated, while a quantitative ratio of <0.67 was considered to be downregulated for the bioinformatics analysis. GO annotation of the proteome was performed using the UniProt-GOA Database (http://www.ebi.ac.uk/GOA/). Proteins were categorized into biological processes and molecular functions according to the GO annotation. The KEGG was utilized to annotate pathways. For functional enrichment analysis, we used Fisher’s exact test to assess the enrichment or depletion (two-tailed test) of specific annotation terms among members of the resulting protein clusters. Any terms with adjusted *p* values below 0.05 in any of the clusters were treated as significant.

### Purification of DosR and its derivative mutants

For the purification of DosR, pET28a-dosR, pET28a-dosR^K182R^, and pET28a-dosRK^182Q^ were constructed and verified by sequencing. All confirmed plasmids were transformed individually into *E. coli* BL21, and recombinant strains were grown in Luria Bertani medium containing 100 μg/mL ampicillin at 37 °C. Isopropyl β-d-1-thiogalactopyranoside was added to the culture at a final concentration of 0.1 mM when the absorbance at 600 nm reached 0.6–0.8. The culture was continuously incubated for 16 h at 18 °C. Afterwards, cells were harvested by centrifugation and stored at −80 °C. Thawed bacteria were resuspended in lysis buffer (50 mM Tris-HCl and 0.5 M NaCl, pH 8.0) and lysed by sonication. Insoluble material was removed by centrifugation for 30 min at 19 000 × *g*. The soluble extract was purified by Ni^2+^ affinity chromatography (Qiagen, Hilden Germany) according to the manufacturer’s instructions. Protein purity was monitored by 12% sodium dodecyl sulfate (SDS)–polyacrylamide gel electrophoresis (PAGE) and Coomassie blue staining. The concentration was determined using a BCA protein assay kit (Thermo Fisher Scientific) with BSA as the standard.

### Anti-DosR- and anti-DosR Ac-K182-specific polyclonal antibody preparation

The 6×His-tagged DosR was purified as previously described^46^ and used to immunize rabbits for the production of anti-DosR polyclonal antibody. Simultaneously, the acetylated Ac-K182 peptide FLAEKTVK(ace)NYVSRL, a 175–188 amino acid of DosR, was coupled with keyhole limpet hemocyanin and used as an antigen to immunize rabbits. The antiserum was collected and purified with acetylated peptide, and control K182 peptide FLAEKTVKNYVSRL was used to remove nonspecific antibodies. The sensitivity and specificity of anti-DosR and anti-DosR Ac-K182 antibodies were evaluated by western or dot blots.

### Construction of plasmids and recombinant *M. smegmatis*

The *dosR*, *phop*, *sigA*, *sigB*, *sigF*, and *lsr2* genes of *Mtb* were amplified using *Mtb* H_37_Rv genomic DNA as a template and gene-specific primers with *Nde*I and *Hind*III enzyme-cutting sites (Supplementary Table S4) were cloned into the *E. coli*/mycobacterium shuttle plasmid, pVV16, and the K-R mutant. Recombinant plasmids were electrically transformed into *M. smegmatis*, and transformants were selected on 7H10 agar containing 10% ADC, 50 µg/mL hygromycin B, and 50 µg/mL kanamycin. Recombinant mycobacteria were further cultured in 7H9 broth supplemented with 10% ADC enrichment, 0.05% Tween 80, 50 µg/mL hygromycin B, and 50 µg/mL kanamycin. *M. smegmatis* harboring the vector backbone, pVV16, was included as a control.

### Rv0998 and DosR mutant constructions in *Mtb*

Gene knockouts of *Rv0998* and *DosR* were generated using allelic exchange and a specialized transducing phage, phAE87 (provided by Dr. LD Lyu, Shanghai Institutes for Biological Sciences)^47^. Constructs for allelic exchange were generated by amplifying the upstream and downstream flanking regions of each of the two genes using the primer pairs listed in Supplementary Table S4. Upstream and downstream flanking regions were cloned into the delivery vector pYUB854. Allelic exchange constructs were incorporated into the phAE87 phage. Phagemid DNA was electroporated into electrocompetent *M. smegmatis* cells, and these were then plated on 7H10 agar plates to obtain plaques at the permissive temperature of 30 °C. Specialized transducing phages were picked and amplified at 30 °C to generate high titer mycobacteriophages. The desired phage was transduced into the exponential phase of *Mtb* H_37_Rv to delete genes of interest by specialized transduction. The transductants were plated on selective medium consisting of 7H10 agar containing 10% ADC enrichment and 75 μg/mL hygromycin and then cultured at 37 °C. Hygromycin-resistant clones were isolated and analyzed by PCR and RT–PCR to confirm the deletion of target genes. Western blotting was used to confirm the deletion of DosR with anti-DosR polyclonal antibody; anti-SigA (Biolegend, San Diego, CA) monoclonal antibody was used as the reference antibody. The complementation plasmid for the *dosR* and K182R mutant was generated by ligating the PCR product into the shuttle vector, pVV16. The resulting plasmid was transformed into the Δ *dosR* strain and plated on a 7H10 plate containing kanamycin at 50 μg/mL. Positive integrants carrying the required insert were screened by colony PCR and validated by RT–PCR.

### Growth curve

Mycobacteria were grown to mid-log phase in 7H9 broth with 10% ADC, 0.05% Tween 80, and antibiotics, as required. Growth curves for each strain were determined using a Bioscreen C Growth Curve Instrument (Labsystems Oy, Helsinki, Finland) and a honeycomb plate with 100 wells (Labsystems Oy)^48^. Briefly, 200 μL of each bacterial suspension, adjusted to a similar density, was added to each well and cultured with shaking at 37 °C. The optical density was measured at an absorbance of 590 nm every 2 h. Hypoxic conditions were established by covering each culture with 50 μL of paraffin oil. Cultures were incubated at 37 °C for 7 days for *M. smegmatis*, and 14 days for *Mtb*. Two independent experiments were performed, each in triplicate.

### Quantitative RT–PCR assay

Bacteria were cultured as previously described, harvested, and disrupted by a bead homogenizer. RNA was isolated using Trizol (Invitrogen). Primers for quantitative PCR are listed in Table S4. Samples were run in triplicate and amplified using real-time SYBR Green quantitative reagents (Toyobo, Osaka, Japan). The relative transcriptional level was determined by the 2^−ΔΔCt^ method. The reference gene was 16S rRNA.

### Electrophoretic mobility shift assays

DNA fragments used for EMSAs were amplified by PCR using *Mtb* H_37_Rv genomic DNA as a template. The promoter region of *acr* was amplified using the primers *acr*-F and *acr*-R, which are listed in Supplementary Table S4. PCR amplification yielded fragments of 148 bp. Approximately 0.25 μg of DNA in a 20 μL volume was incubated at room temperature for 10 min with 5 μg of purified DosR or derivative mutant protein. The binding buffer used for protein–DNA incubations contained 25 mM Tris-HCl (pH 8.0), 20 mM KCl, 0.5 mM EDTA, 6 mM MgCl_2_, 1 mM DTT, and 1 μg/mL poly I:C. Samples were separated on a 6.5% non-denaturing Tris-glycine polyacrylamide gel and detected by silver staining, as described previously^49^.

### Purification of Rv0998

Rv0998 were overexpressed and purified using similar procedure as that for DosR. A plasmid, pET28a-Rv0998/BL21, was constructed and used to overexpress proteins. The 6× His-tagged Rv0998 was purified by Ni^2+^ affinity chromatography, and the amount was estimated by SDS–PAGE and BCA protein assay.

### Immunoprecipitation of DosR

Previously produced polyclonal antibodies to DosR were used to precipitate specific DosR proteins. For preclearing, cell lysates (500 μg) were incubated with normal rabbit IgG for 1 h at 4 °C. Protein A/G beads (Thermo Fisher Scientific) were added to lysates and incubated for an additional 30 min. The beads were removed by centrifugation, and the precleared supernatant was interacted with DosR antibody at 4 °C for 1 h. Protein A/G beads were added to the lysates and incubated overnight. The beads were pelleted at 4 °C, washed five times with PBS buffer, boiled in SDS sample buffer, and subsequently analyzed by SDS–PAGE and western blot.

### Western or dot blots

Standard western and dot blot procedures were used. Purified recombinant protein (2 μg) or cell extracts (50 μg) were separated by 12% SDS–PAGE and then transferred to a polyvinylidene fluoride membrane. The concentrations of the primary antibodies used in the corresponding blot were anti-DosR (1:1000), anti-DosR Ac-K182 (1:1000), and anti-SigA (1:1000). Horseradish peroxidase-conjugated goat anti-rabbit polyclonal antibody was used as the secondary antibody at a 1:5000 dilution.

### In vitro acetylation

Assays were performed in a 20 μL total reaction volume containing 50 mM Tris-Cl (pH 8.0), 100 mM NaCl, 100 μM acetyl-CoA, 2 μg Rv0998, and 2 μg of DosR or K182 peptide as the substrate in the presence or absence of 200 μM cAMP. Reactions occurred at 25 °C for 10 min and were stopped by boiling in SDS sample buffer. They were then analyzed by western blot with anti-DosR Ac-K182 antibody.

### Intracellular CFUs in peritoneal macrophages

Primary peritoneal macrophages were harvested as described previously^50^. Briefly, C57Bl/6 mice were intraperitoneally injected with 4% Brewer thioglycollate medium (Sigma-Aldrich). After 3 days, the mice were sacrificed by cervical dislocation, and the cells were isolated by flushing the peritoneal cavity with 10 mL of RPMI 1640 per mouse. Cells were seeded in 24-well dishes, and non-adherent cells were removed by extensive washing with RPMI 1640. The adherent peritoneal macrophages were used for subsequent experiments. Bacteria were diluted to achieve a MOI of 1 or 2 and then incubated with cells for 3 h at 37 °C in 5% CO_2_. Then, uninfected cells were washed twice, and infected cells were incubated in fresh tissue culture medium containing 50 μg/mL amikacin for 2 h post infection. Infected cells were lysed at 24 h with 0.1% (v/v) Triton X-100 in PBS. The number of viable intracellular bacteria (CFU) was determined by serial dilutions and plating out.

### Mouse infection

Female C57Bl/6 mice (6–8 weeks old) were challenged by i.p. injection with 5 × 10^6^ CFUs per mouse of the indicated bacterial strains as described previously^50^. Mice were housed in a specific pathogen-free animal laboratory before being moved into a biosafety level (BSL)-3 laboratory for *Mtb* infection. Four weeks after infection, the mice were sacrificed, and the bacterial loads in the lungs and spleens were determined by CFU counts.

### CFU assay

The left portions of lung tissues from infected mice and the whole spleen were homogenized separately in 1 mL of PBS. Homogenates in 10-fold serial dilutions were plated on 7H10 agar supplemented with 10% ADC enrichment and incubated at 37 °C. Colonies were counted after 4 weeks.

### Histopathology analysis

Half of each lung was fixed in 4% neutral-buffered paraformaldehyde solution for 24 h. Lung tissue was then embedded within paraffin. A series of sections with a thickness of 4–7 μm were then cut and stained with hematoxylin and eosin by standard methods. The pathology was evaluated by pathologists in a blinded manner.

### Statistical analysis

Statistical significance was determined with an unpaired two-tailed Student’s *t*-test at a *P* < 0.05 level of significance using GraphPad Prism 5.0 software.

### Data availability

The mass spectrometry proteomics data have been deposited to the ProteomeXchange Consortium via the PRIDE partner repository with the dataset identifier PXD008587. Material requests for bacterial experiments should be addressed to X.C.H. (huangxiaochen.1001@163.com) or J.W. (wjtwo@163.com). Material requests for proteomics studies should be addressed to H.Y. (yanghua97065@163.com) or W.S. (shfksw@126.com). Material requests for purified proteins or antibodies should be addressed to Z.H.L. (nllzh@126.com) or F.L. (liufeng@sibs.ac.cn). All other material requests should be addressed to H.Y. (yanghua97065@163.com) or B.X.G. (baoxue_ge@tongji.edu.cn).

## Electronic supplementary material

Supplementary figure S1

Supplementary figure S2

Supplementary figure S3

Supplementary figure S4

Supplementary figure S5

Supplementary Table S1

Supplementary Table S2

Supplementary Table S3

Supplementary Table S4

Supplementary figure legends
