# Supplementary figures and images for: Lysine acetylation of DosR regulates the hypoxia response of Mycobacterium tuberculosis
Source: Emerg Microbes Infect. 2018 Mar 21;7:34. doi: 10.1038/s41426-018-0032-2 (PMC5861037; doi:10.1038/s41426-018-0032-2)

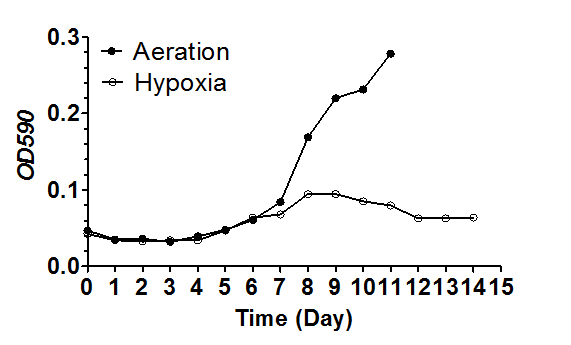

Supplement: Supplementary file 1 — Supplementary figure S1 [file 41426_2018_32_MOESM1_ESM.tif]

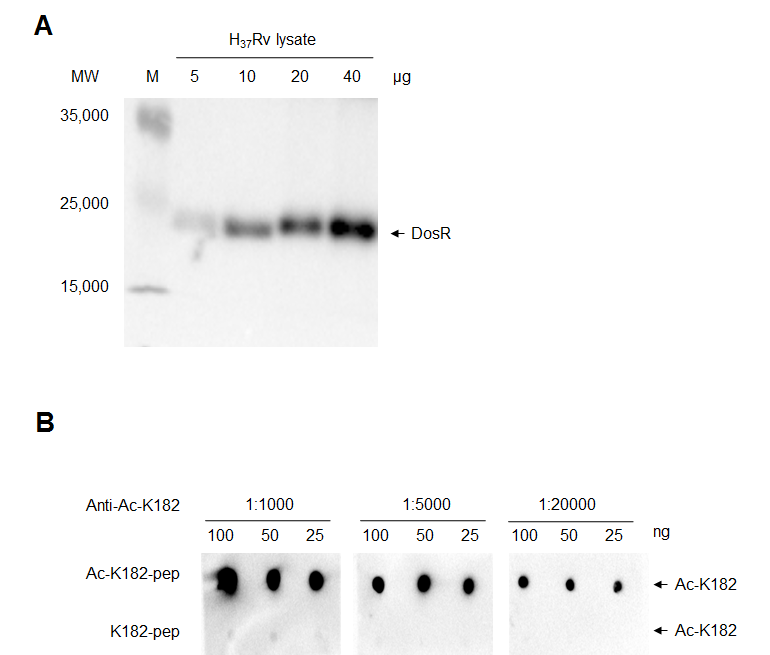

Supplement: Supplementary file 2 — Supplementary figure S2 [file 41426_2018_32_MOESM2_ESM.tif]

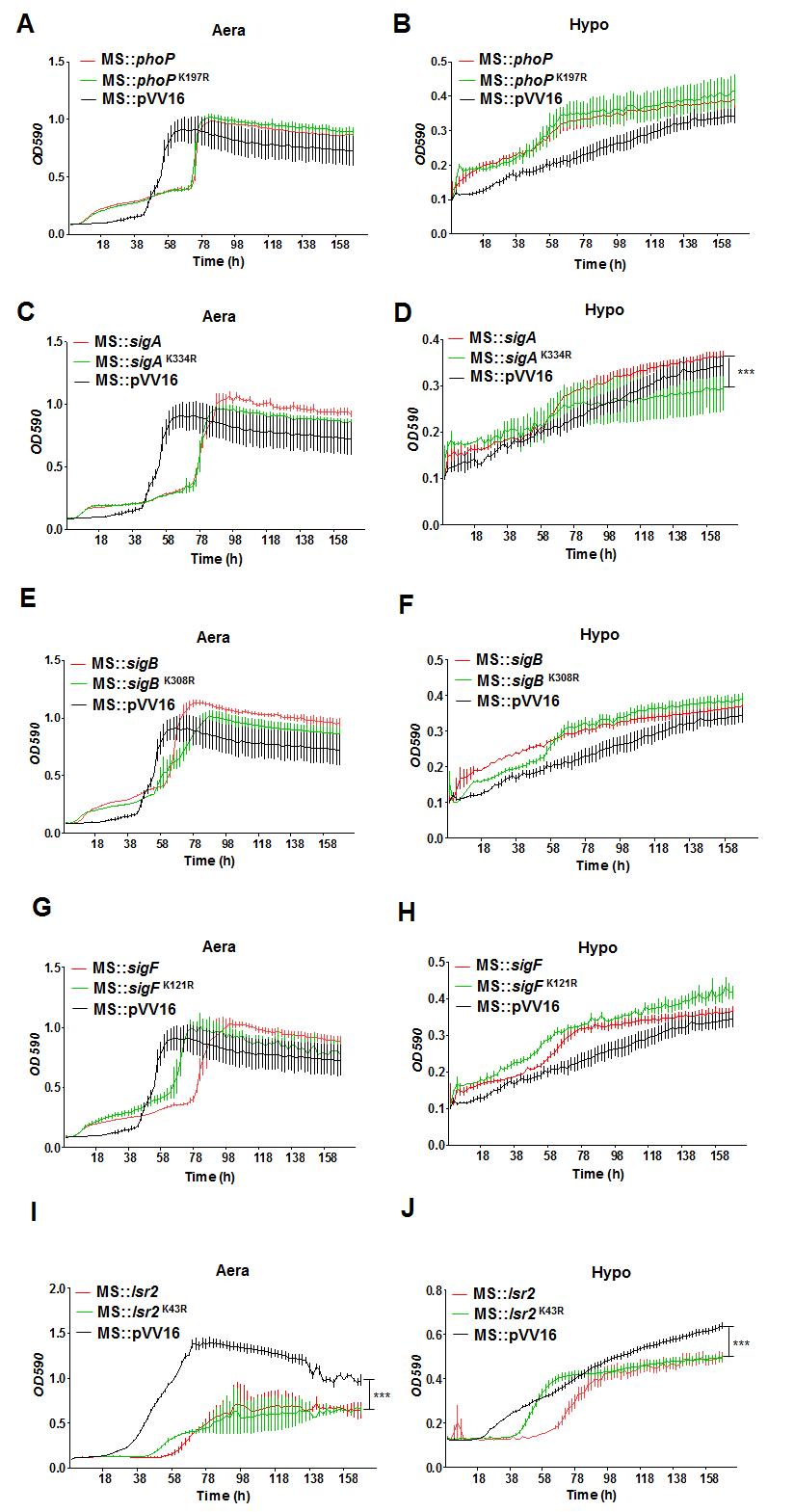

Supplement: Supplementary file 3 — Supplementary figure S3 [file 41426_2018_32_MOESM3_ESM.tif]

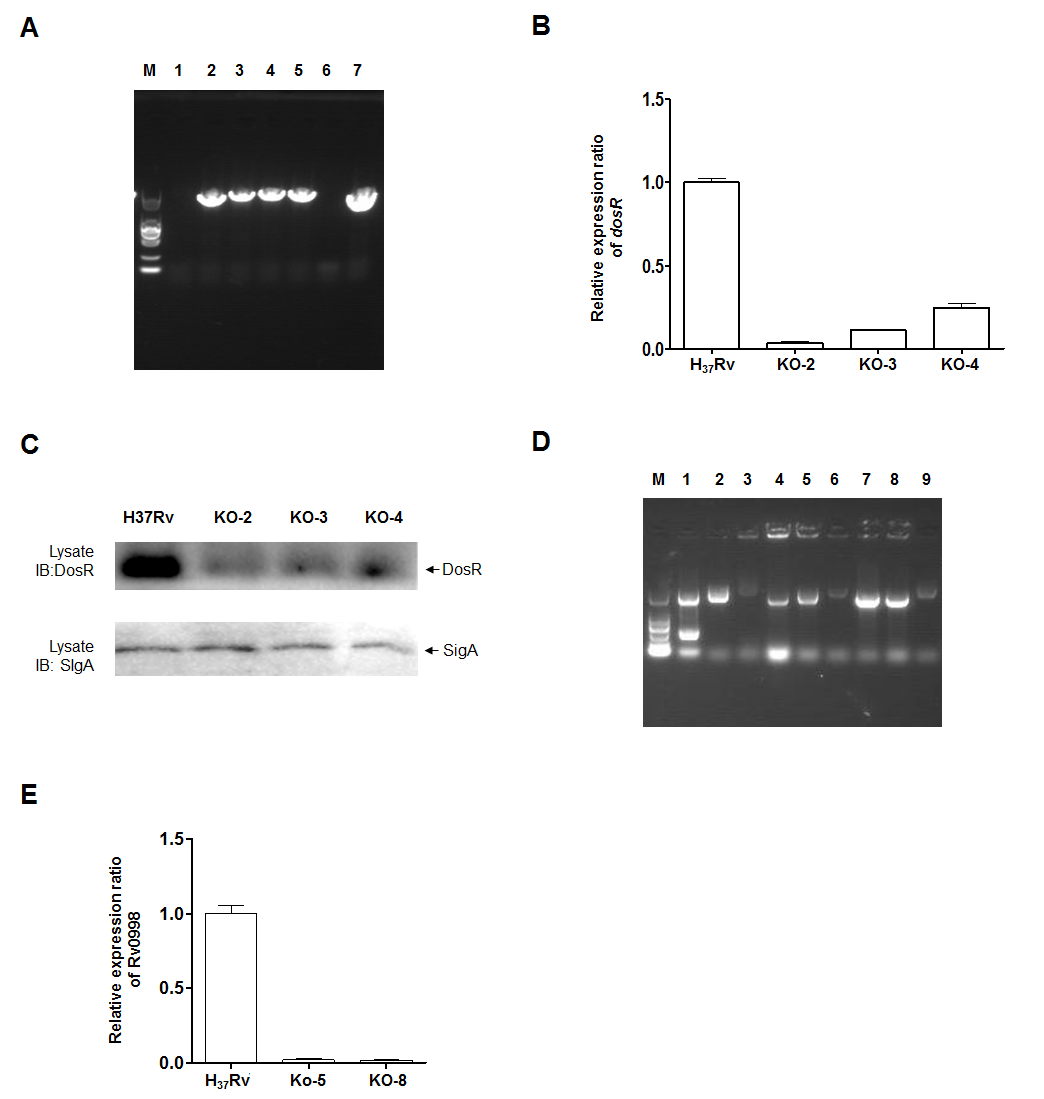

Supplement: Supplementary file 4 — Supplementary figure S4 [file 41426_2018_32_MOESM4_ESM.tif]

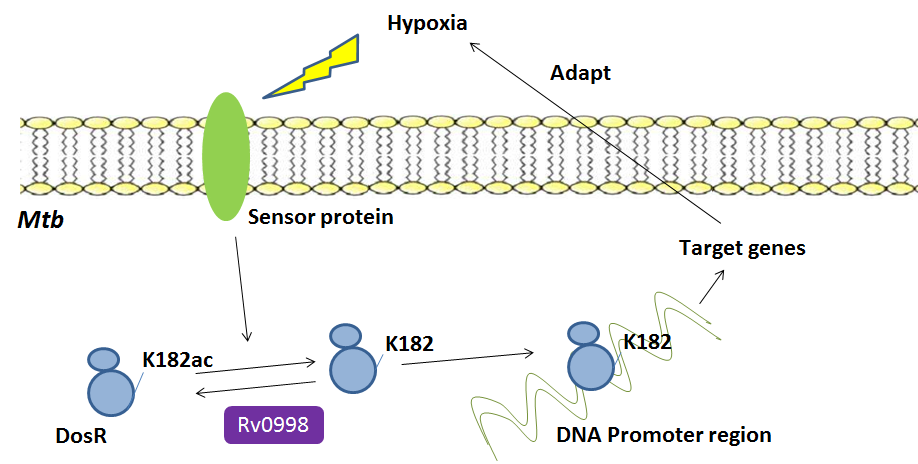

Supplement: Supplementary file 5 — Supplementary figure S5 [file 41426_2018_32_MOESM5_ESM.tif]
