# Supplementary material for: Lysine acetylation of DosR regulates the hypoxia response of Mycobacterium tuberculosis
Source: Emerg Microbes Infect. 2018 Mar 21;7:34. doi: 10.1038/s41426-018-0032-2 (PMC5861037; doi:10.1038/s41426-018-0032-2)
Supplement: Supplementary file 10 — Supplementary figure legends [file 41426_2018_32_MOESM10_ESM.docx]

**Supplementary figure legends**

**Figure S1** Growth curve of *Mtb* H_37_Rv cultured under aeration and hypoxia according to the Wayne model. Nephelo flasks with sidearms was used to culture bacteria. *Mtb* H_37_Rv was grown in 7H9 broth supplemented with 10% ADC and 0.05% Tween 80 at 37°C to mid-log phase (OD580≈0.4). For fully replicating, aerobic control cultures, 200 mL medium was inoculated with 2 mL of the culture in the flask and incubated at 37°C on a magnetic stirrer set to rotate at 180 rpm. Simultaneously, 400 mL medium was inoculated with 4 mL of an identical culture in the tightly-capped flask, which left 200 mL of air space, corresponding to a 0.5 head space ratio (HSR). Then, the tightly-capped flask culture was placed on a tissue culture magnetic stirrer set at 70 rpm and incubated at 37°C. Every day, a portion of the cultures of the two flasks were gently tipped into the side arm to be read at OD590. Samples of the vigorous aeration (Aera) and hypoxic (Hypo) cultures for the quantitative proteomics assay were taken for up to 12 and 14 days, respectively.

**Figure S2** Characterization of anti-DosR and anti-DosR Ac-K182 polyclonal antibodies. Polyclonal antibodies were prepared as described in the “Materials and Methods” section. (**A**) Western blot identification of anti-DosR polyclonal antibody. Cultured *Mtb* H_37_Rv cells were harvested, washed, lysed and then disrupted by sonication. Soluble proteins were collected after centrifugation at 20,000 ×g for 10 min at 4°C. Protein concentrations were determined with a 2-D Quant kit. Serially diluted whole cell lysates were separated by 12% sodium dodecyl sulfate (SDS)–polyacrylamide gel electrophoreses (PAGE) and then transferred to polyvinylidene difluoride (PVDF) membranes. Anti-DosR polyclonal antibody raised by us was used as the primary antibody at a 1:1000 dilution, and horseradish peroxidase-conjugated goat anti-rabbit polyclonal antibody was used as the secondary antibody at a 1:5000 dilution. Western blots are representative of at least three independent experiments. (**B**) Dot blot identification of anti-DosR Ac-K182 polyclonal antibody. Serial diluted Ac-K182 peptides and K182 peptides were dotted on the PVDF membrane. Anti-DosR Ac-K182 polyclonal antibody was used at 1:1000, 1:5000, 1:20000 dilutions, and HRP-conjugated goat anti-rabbit polyclonal antibody was used at a 1: 5000 dilution. Dot blots are representative of at least three independent experiments.

**Figure S3** Growth under aeration and hypoxia of *M. smegmatis* overexpressing five key regulators. We individually overexpressed the five key regulated genes *phoP* (**A, B**), *sigA* (**C, D**), *sigB* (**E, F**), *sigF* (**G, H**), and *lsr2* (**I, J**) from the hypoxia regulatory network in *M. smegmatis*; these were identified as acetylated and varied in response to hypoxic stress. We simultaneously constructed K-R mutant strains of the identified acetylated lysine. Then, the effect of deacetylation on growth, under hypoxia, of the five groups of recombinant *M. smegmatis* was analyzed as previously described. Results shown are of combined data (mean ± SD) from two independent experiments, with each experiment performed in triplicate.

**Figure S4** Characterization of a DosR, Rv0998 knockout *Mtb.* (**A**) An agarose gel showing polymerase chain reaction (PCR) amplification products of DosR deletion clones with *dosR*-KOID primers. Lane M, DL2000 markers; Lane 1, blank control; Lanes 2–6, PCR products from DosR deletion clones 1–5; Lane 7, PCR products from H_37_Rv. The wild-type fragment is 1773 bp, while deletion strains showed the expected 2391 bp fragment. (**B**) Real time (RT)–PCR identification results of DosR deletion clones with *dosR*-RT primers. Total RNA was isolated and transcriptional levels determined by quantitative (q)PCR using the 2^−ΔΔCt^ method. The relative expression of tested *dosR* genes was normalized to that of 16S rRNA. Values represent the mean ± SD from three independent experiments. (**C**) The expression of DosR protein in the deletion mutant. Immunoprecipitation (IP) and immunoblotting (IB) of protein lysates using anti-DosR antibody to detect the expression of DosR in the deletion mutants was performed, with SigA used as the reference protein. (**D**) An agarose gel showing PCR amplification products of Rv0998 deletion clones with Rv0998-KOID primers. Lane M, DL2000 markers; Lane 1, PCR products from *Mtb* H_37_Rv; Lanes 2–9, PCR products from Rv0998 deletion clones 1–8. The wild-type fragment is 1954 bp, while the deletion strains showed the expected 2370 bp fragment. (**E**) RT–PCR characterization of Rv0998 deletion clones with Rv0998-RT primers. Total RNA was isolated and transcriptional levels determined by qPCR using the 2^−ΔΔCt^ method. The relative expression of the tested gene, *Rv0998*, was normalized to that of 16S rRNA. Values represent the mean ± SD from three independent experiments.

**Figure S5** Diagram depicting lysine acetylation of DosR regulates the hypoxia response of *Mtb.* Hypoxia induces the deacetylation of DosR, which in turn increases its DNA-binding ability to promote the transcription of DosR downstream target genes, leading to the rapid adaption to hypoxia of *Mtb*. Rv0998 can acetylate DosR^K182^ and negatively regulate the adaption of *Mtb* to hypoxia.
